# Development of a Bifunctional Ti-Based Gas Diffusion Electrode for ORR and OER by One- and Two-Step Pt-Ir Electrodeposition

**DOI:** 10.3390/nano12071233

**Published:** 2022-04-06

**Authors:** Maximilian Cieluch, Pit Yannick Podleschny, Norbert Kazamer, Florian Josef Wirkert, Ulrich Wilhelm Rost, Michael Brodmann

**Affiliations:** 1 Westphalian Energy Institute, Westphalian University of Applied Sciences Gelsenkirchen Bocholt Recklinghausen, Neidenburger Str. 43, 45897 Gelsenkirchen, Germany; pit.podleschny@w-hs.de (P.Y.P.); florian.wirkert@w-hs.de (F.J.W.); ulrich.rost@w-hs.de (U.W.R.); michael.brodmann@w-hs.de (M.B.); 2 ProPuls GmbH, Neidenburger Str. 10, 45897 Gelsenkirchen, Germany

**Keywords:** bifunctional electrode, unitized regenerative fuel cell, ready to use electrode, electrolysis, fuel cell, Ti-substrate, co- and successive Pt-Ir electrodeposition, wet-chemical etching, micro- and nanostructure

## Abstract

The present paper presents one- and two-step approaches for electrochemical Pt and Ir deposition on a porous Ti-substrate to obtain a bifunctional oxygen electrode. Surface pre-treatment of the fiber-based Ti-substrate with oxalic acid provides an alternative to plasma treatment for partially stripping TiO_2_ from the electrode surface and roughening the topography. Electrochemical catalyst deposition performed directly onto the pretreated Ti-substrates bypasses unnecessary preparation and processing of catalyst support structures. A single Pt constant potential deposition (CPD), directly followed by pulsed electrodeposition (PED), created nanosized noble agglomerates. Subsequently, Ir was deposited via PED onto the Pt sub-structure to obtain a successively deposited PtIr catalyst layer. For the co-deposition of PtIr, a binary PtIr-alloy electrolyte was used applying PED. Micrographically, areal micro- and nano-scaled Pt sub-structure were observed, supplemented by homogenously distributed, nanosized Ir agglomerates for the successive PtIr deposition. In contrast, the PtIr co-deposition led to spherical, nanosized PtIr agglomerates. The electrochemical ORR and OER activity showed increased hydrogen desorption peaks for the Pt-deposited substrate, as well as broadening and flattening of the hydrogen desorption peaks for PtIr deposited substrates. The anodic kinetic parameters for the prepared electrodes were found to be higher than those of a polished Ir-disc.

## 1. Introduction

Radical steps need to be taken with respect to current energy pathways to meet the objectives of the Paris agreement, in particular, the reduction of greenhouse gas emissions across multiple sectors (e.g., transport, industry and building heating) [1]. Therefore, conversion of the energy sector towards power generation based on renewable, fluctuating sources is required [2,3]. Hydrogen, as an efficient and clean energy source [4,5], can become a possible energy carrier of the future [6], with an expected sharp decrease in implementation costs [7,8].

Unitized regenerative fuel cells (URFCs) can be operated as either fuel cells or electrolysers; they consist of one electrochemical cell equipped with bifunctional electrodes, resulting in a round-trip energy converting device [9,10]. The operating temperature, and the type of electrolyte (e.g., polymer electrolyte, alkaline and solid oxide), are the main characteristics that differentiate cells [9,11,12]. The most common URFC technology is proton-exchange-membrane (PEM)-based, reaching round-trip energy efficiencies of 35%, whereas solid oxide-based URFCs reach 45% [13,14,15]. In comparison, PEMWEs reach efficiencies in the range of 60–80% [16] and PEMFCs reach 50–70% [17]. The efficiency of PEM-based URFCs suffers from sluggish reaction kinetics at the oxygen electrode and mass transport limitations of gaseous reactants to the active sites [11]. 

High membrane electrode assembly (MEA) costs represent a bottleneck for upscaling PEM-based hydrogen technology [18,19,20,21]. Combining both hydrogen technologies, the UR-PEMFC offers the possibility of cost savings due to the use of one electrochemical cell consisting of electrodes with bifunctional catalyst layers [10,22]. Further cost reduction is achievable by reduction in the noble metal catalyst loading on the electrodes for PEM-based hydrogen energy systems [23,24,25,26,27]. 

The performance of URFCs is dependent on the configuration of the oxygen electrode and influenced by catalyst preparation, deposition technique and deposition order [9,11]. Beginning with the electrode substrate, Ti-based or Ti-reinforced materials, such as M-TiO_2_, Ti_n_O_2n−1_, TiC or TiCN [9], were recently investigated by different research groups [28,29,30,31,32]. In contrast, carbon-based materials suffer from carbon-corrosion due to the anodic potentials (>1.5 V), humidity, temperature, and the presence of oxygen during WE-mode [9,10].

The bifunctional catalyst layer of the oxygen electrode catalyzes the oxygen evolution reaction (OER) during WE-mode, and the oxygen reduction reaction (ORR) during FC-mode [33]. PtIr is a dual function catalyst system able to catalyze both reactions on the oxygen electrode [9,10,34]. To reduce the noble metal catalyst loading, advanced methods, such as hydroxide nanosheet decorations or perovskite structure synthesis, have recently been reported [35,36]. Further, Zhang et al. used a one-pot chemical synthesis method to prepare Ir@Pt nanodendrites as bifunctional oxygen electrode catalysts [37]. Electrochemical characterization of these catalyst structures showed promising results in a three-electrode setup, using a highly conductive glassy carbon rotating disc electrode (RDE) [35,36,37]. Although validating the fundamental principle, as so often, the catalysts were not incorporated into application-relevant assemblies to demonstrate meaningful industrial performance [38]. Du et al. compared mixtures of conventional PtIr alloy nanoparticles and PtIrO_2_ nanocomposites, immobilized onto a commercial carbon support [39]. In addition, Jung et al. investigated a dual function PtIr catalyst on a carbon support by physical mixture in varying proportions, resulting in a 19–36% round trip efficiency for URFCs at 1 A cm^−2^ [40]. Nevertheless, all these combinations would suffer from carbon corrosion caused by anodic potentials during WE operation and therefore not being considered feasible in application-oriented investigations.

Catalyst support materials based on carbon have also been considered, but the use of these supports is not favored at the oxygen electrode due to carbon corrosion [9,10]. Ceramic materials, such as titanium sub-oxides (for example, Ti_4_O_7_ or Ti_5_O_9_), or metal oxides, such as antimony doped tin-oxide (ATO), have been investigated to substitute carbon-based catalyst supports. Nevertheless, commercial ATO offers less specific surface area compared to Vulcan XC-72, resulting in 1.7 m^2^ g^−1^ and 205 m^2^ g^−1^, respectively [9]. Therefore, Cruz et al. synthesized ATO with an increased specific surface area of 115.17 m^2^ g^−1^ using a colloidal method, leading to a round-trip efficiency of 48% at low current densities for the URFC [41]. Another approach, chosen by Huang et al., was the use of titanium supported Pt and Ir as the electrocatalyst, combined with a carbon-based substrate covered by an IrTi-nitride micro-porous layer [31]. These bifunctional oxygen electrodes led to a round-trip efficiency of 42%, whereas the unsupported PtIr catalyst achieved only 30% round-trip efficiency [31]. Modified catalyst support structures are advanced scientific developments that undoubtably enable impressive physicochemical properties. Nevertheless, the integration of such advanced catalyst synthesis methods into a cost-efficient electrode manufacturing chain represents an “elephant in the room” type of problem that is too little scientifically challenged.

To summarize, recent research into bifunctional electrochemical catalysts, support materials and electrode substrates utilizes complex, laboratory-scale methods of synthesis. Electrochemical catalyst deposition can be considered an easy, fast, scalable and industrial preparation process for electrode manufacturing. Moreover, electrochemical catalyst deposition, compared to the state-of-the-art applied spray-coating approach of chemically synthesized catalyst particles, offers the advantages of increased catalyst utilization, as the catalyst can be deposited as a graded catalyst layer [42,43], and the suppression of supporting material corrosion due to mechanical connection of the catalyst to the electrode substrate [10]. Moreover, suppression of performance losses caused by cracking and dissolution of the ionomer binder [10] is achievable, due to the process-related graded catalyst layer. Hence, the catalyst particles may be connected directly to the solid or liquid electrolyte. Furthermore, the use of a catalyst support structure has been replaced by wet-chemical acid pre-treatment of the Ti-substrate, which offers further cost reduction as a result of expensive support structures and associated working steps substitution. 

In the present study, the deposition of noble metal catalysts on a Ti-substrate was investigated to prepare a bifunctional catalyst layer using an electrochemical deposition method. Here, the ex-situ characterization of a Ti-based electrode with a Pt catalyst is first presented, followed by investigations regarding the bifunctional, electrochemical co- and successive deposited PtIr catalyst layer. The highly porous substrate material provides a large specific surface area and resistance to physiochemical and electrochemical corrosion. The characteristics of the material overcome some of the important challenges of an URFC. Combined with the fact that research is focusing on the investigation of ready-to-use electrodes, the results can be considered as valuable input for industrial scaled implementation. The bifunctionality of electrodes is strongly linked to factors such as particle size, distribution, and their interaction with the support material. All these aspects are thoroughly discussed in the present paper. 

## 2. Materials and Methods

### 2.1. Electrode Preparation

#### 2.1.1. Substrate Cleaning

**Figure 1:**
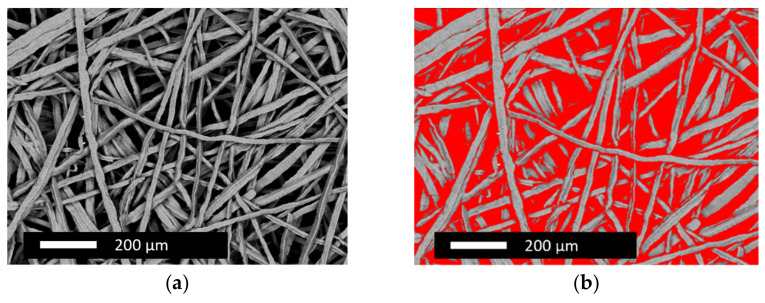
Topography of the (**a**) untreated and (**b**) the calculated porosity Ti-substrate showing its fiber structure and porosity.

As the electrode substrate, a commercial Ti-based fleece material from BEKAERT, Zwevegem, Belgium, (Bekipor^®^ ST 2 GDL-40-1.5) was chosen with a Ti-fiber diameter of 22 × 10^−6^ m and an approximate porosity of 70% according to the material datasheet. Figure 1 shows the untreated Ti-substrate offering an increased geometrical surface area due to the fiber structure and therefore supporting the electrochemical catalyst deposition. The high porosity of the substrate is beneficial for the water management of the electrode. The water produced during FC-mode can be easily removed from the catalyst layer and supplied during WE operation.

Ti-substrates with a geometric area of 1 cm^2^ were initially cleaned from processing residues in an ultrasonic bath using deionized water (Direct Q^®^ 3 UV, Merck Group, Darmstadt, Germany), acetone (ROTIPURAN^®^ min. 99.8%, CarlRoth, Karlsruhe, Germany) and ethanol (Ethanol absolut ≥99.5%, GPR RECTAPUR^®^, CarlRoth, Karlsruhe, Germany) for 10 min each. To enable electrical contact for the electrochemical catalyst deposition, and to provide deposition sites for Pt and Ir, stripping of the highly stable and electrical insulating TiO_2_ from the substrate surface was necessary. Therefore, two surface etching processes were investigated—physical etching with argon plasma was compared to wet-chemical etching with 10% *w*/*v* oxalic acid aqueous solution (hereafter referred to as oxalic acid).

Physical etching was performed based on previous research [44,45,46] using higher energy micro-frequency (mf)-plasma for 10 min in Ar atmosphere with a gas debit of 100 mL min^−1^, and a chamber pressure of 60 Pa at 400 W using a PINK V15-GM device from Pink GmbH Thermosysteme, Wertheim, Germany. The wet chemical etching, adapted based on previous research on similar substrates [47,48,49], was performed for 30 min in 10% *w*/*v* oxalic acid aqueous solution (Alfa Aesar, Haverhill, MA, USA) at 80 °C. 

**Figure 2:**
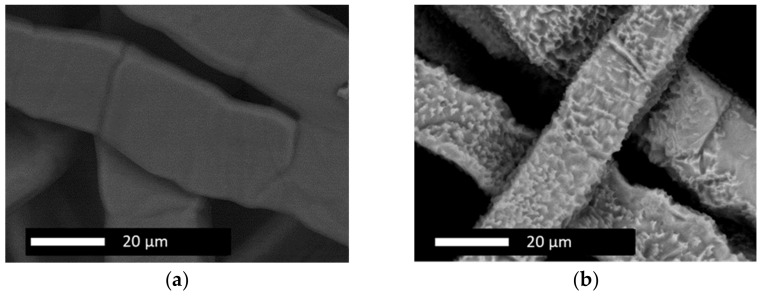
Topography of Ti-substrates after (**a**) plasma and (**b**) oxalic acid treatment.

The topography of the plasma-treated Ti-fibers (as shown in Figure 2a) remained unchanged in comparison to the untreated substrate shown in Figure 1. This result is in good agreement with the findings reported for radio-frequency-plasma-treated samples [46]. No notable difference in the oxygen quantification between plasma and acid-treated substrates could be observed by energy dispersive emission spectroscopy (EDX). As seen in Figure 2b, the oxalic acid treatment roughened the Ti-fiber topography resulting in an increased specific surface area, which favored electrochemical catalyst deposition. Thus, oxalic acid treatment was chosen for the present research, eliminating an expensive step in the electrode preparation chain.

#### 2.1.2. Electrochemical Catalyst Deposition

After wet-chemical etching, the Ti-substrate was rinsed with deionized water and then directly immersed into the working electrode of the galvanic cell for electrochemical noble metal deposition. A galvanic cell with a two-electrode configuration and an IVIUM Vertex potentiostat were used for electrodeposition. All Ti-substrates were cleaned with deionized water after each electrochemical deposition to remove electrolyte residues.

The Ti-based electrode with single Pt catalyst was obtained by a combined deposition process, applying the deposition parameters displayed in Table 1 at 55 °C bath temperature. First, Pt was deposited via a constant potential deposition (CPD) method followed by switching the deposition mode to pulsed electrodeposition (PED). During CPD the potential was registered at the counter electrode (CE) according to Mitzel [50]; therefore, the potential in this work is named vs. CE. The process was performed using a platinized Ti counter electrode (WIELAND Edelmetalle GmbH, Pforzheim, Germany) and a modified Pt electrolyte (Galvatron Platinbad, WIELAND Edelmetalle GmbH, Pforzheim, Germany) based on H_2_PtCl_6_ with a Pt content of 2.5 g L^−1^.

**Figure 3:**
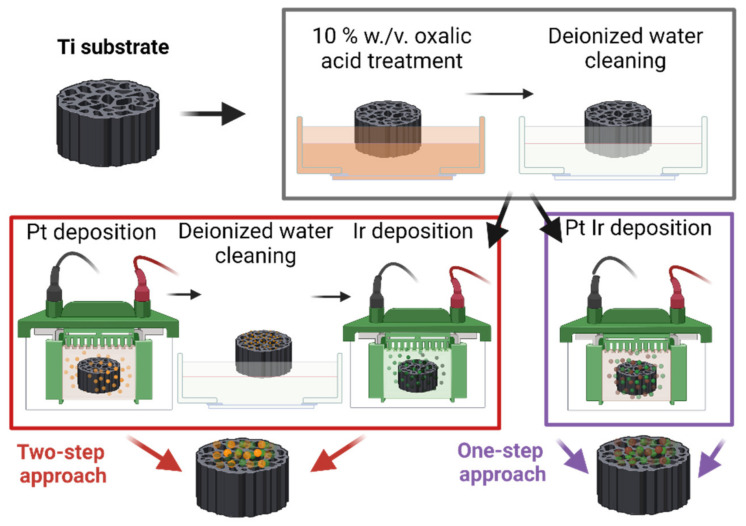
Schematic process of the electrode preparation chain, showing the one-step and two-step PtIr deposition method (co- and successive PtIr deposition).

The two-step PtIr deposition was based on two different electrolytes and galvanic cell configurations. First, the Ti-substrate was deposited with Pt according to the previously presented deposition method. Subsequently, Ir was deposited on the Pt decorated Ti-substrate via the PED method, as schematically depicted in Figure 3, applying the deposition parameters from Table 1 at 85 °C bath temperature. The galvanic cell was equipped with an IrRu-mixed-oxide counter electrode (Industrie De Nora S.p.A., Milan, Italy) and Ir electrolyte (Preciousfab Ir300 ready to use, Metalor Technologies SA, Marin, Switzerland) with an Ir content of 15 g L^−1^. 

The one-step deposition of PtIr catalyst was performed using the PED method, applying the deposition parameters from Table 1 at 90 °C bath temperature. An Ir/Ru-mixed-oxide counter electrode (Industrie De Nora S.p.A., Milan, Italy) and a binary alloy PtIr-electrolyte (STEC GmbH, Mittweida, Germany) were used. The Pt-Ir electrolyte was based on H_2_PtCl_6_ and Br_6_IrNa_3_ dissolved in deionized water, serving as the metal ion source with a concentration of 26 mmol L^−1^ Ir and 2.5 mmol L^−1^ Pt, respectively.

### 2.2. Physical Characterisation

Micrographs were collected with three different scanning electron microscopes (SEMs). A Philips XL 30, Eindhoven, The Netherlands, was used to acquire the micrographs for the substrate analysis at 10 mm working distance and a cathode voltage of 25 kV and was equipped with an FEI energy dispersive X-ray spectroscopy (EDX) detector, Hillsboro, Oregon, United States, for investigation of the chemical composition and catalyst distribution. In addition, JEOL JSM-7500F, Tokyo, Japan, and TESCAN CLARA GMH HighVac UHR FE-REM equipment, Brno, Czech Republic, was used to acquire high resolution micrographs of the deposited catalyst structures, both equipped with an Octane Elect Plus detector from EDAX AMETEK, Mahwah, New Jersey, United States for EDX investigations.

Depending on the total catalyst loading of the electrodes, atomic absorption spectroscopy (AAS), or inductive-coupled-plasma optical emission spectroscopy (ICP-OES), was performed to determine the specific noble catalyst loading. For AAS, a ContrAA800 apparatus from AnalytikJena, Jena, Germany, and for ICP-OES, Spectro Aros equipment from Spectro, Kleve, Germany, were used, respectively. The exact working parameters cannot be provided, these being confidential to MikroLab-Kolbe.

### 2.3. Electrochemical Characterisation

Electrochemical measurements were performed in a three-electrode cell configuration in 0.5 sulfuric acid on an IVIUM Vertex potentiostat. The electrolyte was de-aerated by nitrogen, using a flow rate of 100 mL min^−1^ for 30 min. During the measurements, the cell was continuously purged with nitrogen at a flow rate of 10 mL min^−1^. The experiments were conducted at room temperature on substrates exposing a 1 cm^2^ geometrical surface area serving as the working electrode. A reversible hydrogen electrode (RHE) was used as the reference electrode and a coiled Pt wire as the counter electrode. The cyclic voltammograms were analyzed after 200 cycles to ensure the complete removal of impurities at the catalyst surface at a scan rate of 100 mV s^−1^, performed between 0.05 V vs. RHE and 1 V vs. RHE for the Pt deposited electrodes, and 1.4 V vs. RHE for the PtIr deposited electrodes, respectively. The kinetic parameters were calculated from the linear voltammograms attained with a scan rate of 5 mV s^−1^ between 0.05 V vs. RHE and 1.6 V vs. RHE.

## 3. Results and Discussion

### 3.1. Ti-Based Electrodes with Single Pt Catalyst

**Figure 4:**
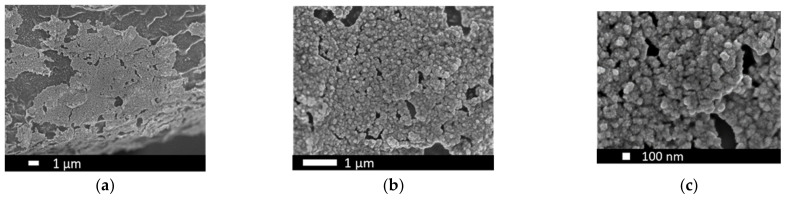
SEM-micrographs of Pt agglomerates obtained via a combined electrochemical deposition method onto Ti-substrate at (**a**) 5000×, (**b**) 20,000× and (**c**) 50,000× magnification.

Figure 4 shows SEM micrographs of the Pt decorated Ti-fibers. In Figure 4a, it can be seen that the fibers were partly covered with Pt agglomerates. In the areas where less Pt was deposited, the surface modification of the Ti-fibers due to the wet-chemical etching process can be seen, which is already evident in Figure 2b. Due to the two-step deposition process of the Pt, the agglomerates consisted of areal, macro-scaled clusters (Figure 4a), covered with nanosized Pt agglomerates (Figure 4c). The areal, macro-scaled Pt agglomerates resulted from CPD. Due to the lower current density compared to PED less growth-nuclei were obtained, resulting in increased nuclei growth [50]. The smaller Pt agglomerates on top of the areal sub-structure (Figure 4c) resulted from increased current density in the PED process, leading to an increased number of growth-nuclei and, therefore, smaller agglomerates. Similar Pt structures have previously been reported by our research group, using different pre-treatment and deposition parameters [43]. Moreover, the obtained deposition-dependent Pt agglomerate structures were in good agreement with the characterizations of Abraham et al. [51].

**Figure 5:**
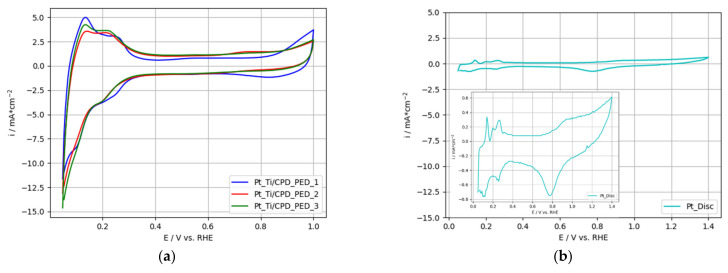
Cyclic voltammograms of (**a**) Ti-based electrodes with electrochemical deposited Pt, (**b**) polished, high purity Pt-disc.

Cyclic voltammograms from several electrodes manufactured under the same pre-treatment and Pt-deposition procedure and a polished, high-purity Pt-disc (1 cm^2^ geometrical area) as reference, were analyzed for their ORR activity and are presented in Figure 5a,b, respectively.

The above outlined Pt agglomerate structure of the Pt_Ti/CPD_PED-electrodes provided an enhanced electrochemical active surface area compared to bulk Pt (Figure 5b). The hydrogen ad- and desorption peaks of the Pt catalyst were clearly evident around 0.05 V vs. RHE up to 0.3 V vs. RHE. Similar peak locations were reported by Abraham et al. [51] for electrodeposited Pt on Ti-substrate measured in 0.5 M H_2_SO_4_. 

To compare the electrodes with respect to electrochemical ORR activity, the electrochemical surface areas (ECSAs) were calculated (Table 2). To obtain an ECSA, the hydrogen desorption charge (Q_Desorp_) was calculated by numerical integration of the hydrogen desorption peaks and divided by the specific charge of a monolayer of hydrogen adsorbed on a Pt surface, leading to the surface roughness factor (SRF). The specific charge for a smooth, polycrystalline Pt surface was set to 210 µC cm^−2^ and is widely used to determine the true (microscopic) surface area of Pt electrodes. The ECSA was obtained by dividing the SRF with the catalyst loading of the electrode. The specific Pt loading of the Ti-based electrodes was determined via AAS, the results being listed in Table 2 [51,52,53].

Although the discussed Pt_Ti/CPD_PED-electrodes were prepared under the same conditions, a variance in the characterization results can be observed. The specific Pt loadings for the electrodes deviated by a maximum of 64.7 µg cm_geo._^−2^. The cyclic voltammograms also indicated a small variation in the formation of the hydrogen peaks, which may have resulted from the different Pt loadings.

The divergence can be attributed to the wet-chemical pre-treatment of the Ti-substrate, which modifies the topography of the Ti-fibers selectively. This might create different specific surfaces of the Ti-substrates, resulting in different current densities (normalized on the specific surface area) during electrochemical catalyst deposition, leading subsequently to different Pt agglomerate structures. These can affect the ORR activity in the cyclic voltammograms. In addition, TiO_2_ formation during substrate assembly into the galvanic cell can influence the Pt deposition and, therefore, the obtained results.

Comparing the ECSA values where the SRF was normalized to the Pt loading, the variation between the electrodes was a maximum of 0.8 m_echem._^2^ g^−1^, so that the preparation chain can be considered as reproducible to a good approximation.

The ECSA values obtained for the Pt/Ti_CPD_PED electrodes were six to ten times lower than the ECSA for state-of-the-art commercial Pt/C catalysts which range from 60 to 110 m_echem._^2^ g^−1^ [54]. Furthermore, compared to the 83.1 m_echem._^2^ g_Pt_^−1^ and 74.8 m_echem._^2^ g_Pt_^−1^ for ECSA values reported by Du et al. for self-synthesized 20 wt.% Pt/C and 40 wt.% Pt/C, respectively, the Pt/Ti_CPD_PED electrodes were outperformed [39]. It should be noted that the Pt/C catalysts were immobilized onto a highly electrically conductive carbon support. In contrast, the electrodes prepared in this investigation were based on a Ti-fiber substrate. Huang et at. performed a chemical particle synthesis of Pt onto a TiO_2_ support, reaching an ECSA of 30.5 m_echem._^2^ g^−1^ [31]. This was nearly three-times higher than the ECSA values reported here. This may be linked to the small Pt particle size of 4.2 nm characterized by Huang et al., offering high accessibility for electrochemical reaction sites [31]. In contrast, the electrodeposited structures in this investigation consisted of macro- and nanosized Pt agglomerates (see Figure 4).

Abraham et al. electrodeposited Pt onto a Ti-foil, applying different deposition methods. ECSAs in the range of 20 to 2.8 m_echem._^2^ g^−1^, 14 to 2.5 m_echem._^2^ g^−1^, 17.8 to 11.4 m_echem._^2^ g^−1^ and 25.1 to 14.1 m_echem._^2^ g^−1^ were reported for potentiostatic, galvanostatic, cyclic and pulsed deposition methods applying different deposition parameters [51]. The ECSAs in this investigation ranged from 10.3 to 11.1 m_echem._^2^ g^−1^, and were, therefore, to a good approximation, comparable to these values. Nevertheless, the difference in deposition parameters, electrolyte composition and deposition methods should be considered.

### 3.2. Ti-Based Electrodes with Two-Step Deposited PtIr Catalyst

One approach to prepare a bifunctional oxygen electrode chosen was to deposit Pt and Ir separately in a two-step deposition process. More precisely, the previously discussed Ti-based electrode with single Pt catalyst was complemented with subsequent electrochemical Ir deposition. A detailed characterization of the single Ir deposition on the Ti-substrate can be found in a previous publication of our working group [55].

**Figure 6:**
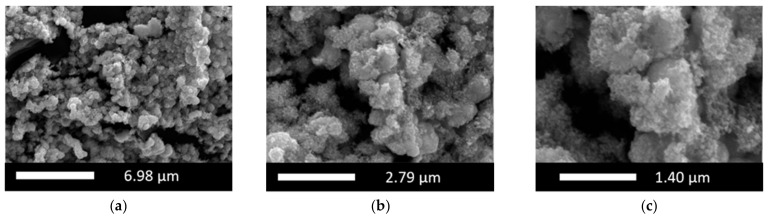
SEM-micrographs of two-step electrodeposited PtIr electrodes at (**a**) 5000×, (**b**) 20,000× and (**c**) 50,000× magnification.

A first insight into the two-step deposited PtIr electrodes (2-PtIr electrodes) is provided by SEM-micrographs in Figure 6. The PtIr catalyst distribution on top of the Ti-fibers shows a similar distribution to that seen in Figure 4a, which is likely to be a result of the initial deposition of Pt with no variation in parameters and the subsequent Ir deposition. Close examination shows that the Ti-fibers were partly covered with PtIr nano agglomerates, meaning that the subsequent deposition of Ir did not take place preferentially on the Ti-fibers. EDX mapping of the catalyst layer revealed that Ir was largely found on the Pt sub-structure, not on the Ti-fibers in the analyzed areas, indicating that the Pt covered areas of the Ti-fibers were more electrically conductive than the Ti-substrate due to the TiO_2_ stripping during material pre-treatment. It seems that the formed electric field lines should preferentially conduct to the Pt sub-structure rather than to the Ti-fibers because there was less distance between the sub-structure and the counter electrode of the galvanic cell in the microsize region. However, in order to validate this supposition, the electric field distribution during the catalyst deposition should be analyzed in further studies. 

The SEM-micrographs presented in Figure 6b,c show bright Ir agglomerates on top of the previously mentioned Pt structure (see Section 3.1).

**Figure 7:**
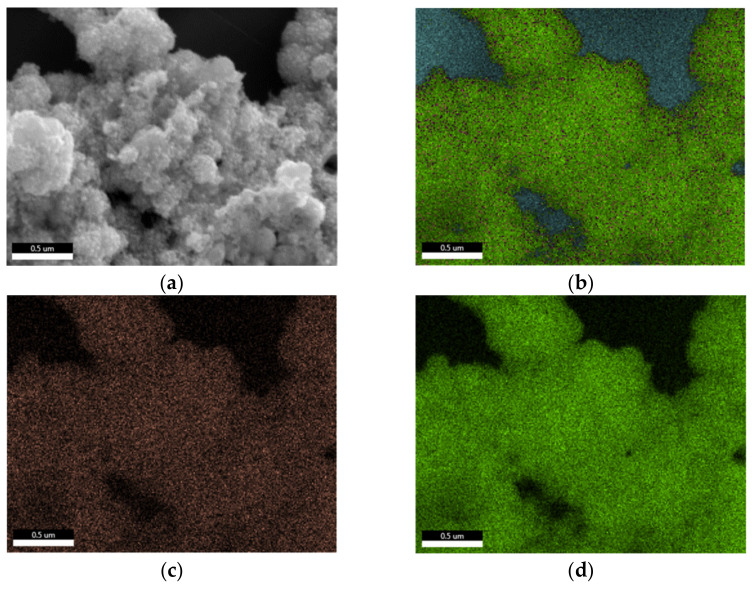
EDX mapping of a two-step deposited PtIr electrode, where (**a**) shows the selected area for the analysis, (**b**) the superposed element analysis, (**c**) the Ir specific analyze and (**d**) the Pt specific analyze.

For quantifying the distribution of catalyst materials, an EDX mapping is presented in Figure 7, revealing a surface element composition of the electrode of 54 wt.% Pt, 28 wt.% Ti and 12 wt.% Ir.

The element-specific micrographs show that the Ir was homogeneously distributed on top of the analyzed Pt agglomerates. Ir clusters were not detected in the analyzed areas of the electrode, the result being supported by the SEM-micrographs.

To determine the catalyst loading, as well as the catalyst composition of the 2-PtIr electrode, AAS was performed for Pt determination, and ICP-OES for Ir determination, respectively. Results are listed in Table 3 showing a weight-related Pt:Ir ratio of 99.77:0.23 wt.%. Due to the chosen CPD followed by PED for the Pt deposition, the catalyst layer agglomerates were mainly composed of Pt. Considering the PED deposition parameters for Ir, the low proportion determined via ICP-OES indicates poor efficiency for the catalyst deposition process.

#### 3.2.1. ORR-Activity Results for Two-Step Deposited PtIr Electrodes

**Figure 8:**
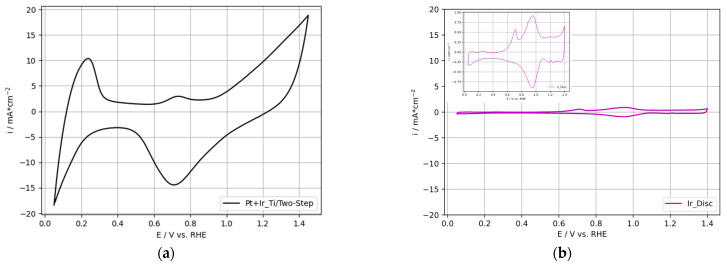
Cyclic voltammogram of (**a**) Ti-based electrode with two-step deposited PtIr, (**b**) polished, high purity Ir-disc (also shown in inset).

Figure 8a presents the cyclic voltammogram of the 2-PtIr electrode; Figure 8b depicts a high purity, polished Ir-disc (1 cm^2^ geometrical area) as reference. Refer to Figure 5 for the cyclic voltammograms of the single Pt deposited on Ti-substrate, as well as the Pt-disc reference.

The cyclic voltammogram obtained for the 2-PtIr electrode revealed higher specific current densities over the entire potential range compared to the Pt-disc, Ir-disc and single Pt electrode. In the voltage range of 0.05–0.3 V vs. RHE, the hydrogen desorption peaks were not separately formed compared to the Pt-disc reference. Instead, one hydrogen desorption peak was obtained without distinct peaks linked to specific oxide transition states. Similarly, anodic sweeps for PtIr catalysts were reported by Petrossians et al. [56]. In the potential range of 0.6–0.8 V vs. RHE, a characteristic Ir peak was shown in the cyclic voltammogram of the 2-PtIr electrode and the Ir reference. This characteristic peak originated either from the oxidation of the Ir(III)-sites near the metal/oxide interface [57] or the chemisorption of O/OH-groups onto the Ir surface [58].

To characterize the ORR activity of the 2-PtIr electrode, the ECSA was calculated (Table 3). Q_Desorp_ was estimated by numerical integration of the anodic hydrogen desorption peak and subsequently divided by the specific charge of a monolayer hydrogen adsorbed on a Pt or Ir surface leading to the SRF. The specific charges for smooth Ir and Pt surfaces covered with a hydrogen monolayer were 217 µC cm^−2^ and 210 µC cm^−2^, respectively. The specific charge values were rated according to the mass specific electrode loading [53].

The specific Pt and Ir loading of the Ti-based electrodes displayed in Table 3 were determined via AAS and ICP-OES, respectively.

The calculated SRF value for the 2-PtIr electrode was 10,657.1 µC cm_geo._^−2^. This was about two-times higher compared to the SRF value for the single Pt electrodes (Table 2). Both electrodes were prepared applying the same Pt deposition procedure and parameters—only the 2-PtIr electrode was supplemented by Ir deposition via PED. 

Topalov et al. [59] and Radev et al. [60] also reported an increased SRF value for ORR by incorporating small amounts of Ir into the Pt catalyst. Nevertheless, taking into consideration the specific Pt and Ir loadings of the 2-PtIr electrode (Table 3), the increased SRF value can be directly linked to the increased Pt mass determined via AAS. The Pt mass of 2470.3 µg cm_geo._^−2^ was nearly ten-times higher compared to the single Pt electrodes, leading to the low ECSA value of 2.1 m_echem._^2^ g^−1^. 

The discrepancy in catalyst loadings between 2470.3 µg cm_geo._^−2^ for the 2-PtIr electrode compared to 197.8 µg cm_geo._^−2^ –262.5 µg cm_geo._^−2^ for the single Pt electrodes, applying equal parameters and procedures for the electrochemical Pt deposition, may have resulted from the CPD sub-step of the deposition process. In the literature, a three-electrode configuration for CPD deposition is recommended to maintain the potential at the phase boundary of the electrolyte/electrode [50,61]. In this investigation, the CPD was performed in a two-electrode configuration. Therefore, it is possible that, during CPD, the applied current was transposed partly into hydrogen due to the hydrogen evolution reaction. Furthermore, the impact of the wet chemical pre-treatment with oxalic acid may have influenced the deposition process by varying the specific surface of the Ti-electrode. In addition, the electrical conductivity of the Ti-substrate prior to the CPD, which was applied first after wet-chemical pre-treatment, can affect the voltage drop across the working electrode and therefore the Pt deposition.

Further in-depth catalyst loading measurements should be obtained by performing a high number of measurements to validate the present investigations. In terms of concreate measures, at least the CPD sub-step of the electrochemical Pt deposition applied in this investigation needs to be performed in a three-electrode configuration to maintain the working electrode voltage, and, therefore, to obtain improved reproducibility of the Pt loadings and results.

Comparing the ECSA of the 2-PtIr electrode to a state-of-the-art ORR Pt/C catalyst, offering an ECSA of 60 m_echem._^2^ g^−1^to 80 m_echem._^2^ g^−1^, the 2-PtIr electrode was outperformed [39,54]. Moreover, for nanocomposite PtIrO_2_/C catalysts prepared via chemical synthesis, ECSAs of 77 m_echem._^2^ g^−1^ and 82.4 m_echem._^2^ g^−1^ have been reported for Pt:Ir ratios of 20:20 wt.% and 20:40 wt.%, respectively, which outperform the 2-PtIr electrode in this study [39]. The discussed catalyst differential in the catalyst supports, the references are carbon-based, while the 2-PtIr electrode is Ti-fiber based. Nevertheless, it should be taken into consideration, that the carbon-based catalysts are less appropriate for application in bifunctional oxygen electrodes due to the carbon corrosion of the catalyst support. Further, Kong et al. prepared a Pt/IrO_2_ catalyst via a chemical reduction method with a molar ratio of 1:1 [62]. Values of 26.9 m_echem._^2^ g^−1^ and 31.8 m_echem._^2^ g^−1^ ECSA were reported for Pt supported on commercial IrO_2_ and self-synthesized porous IrO_2_ [62]. In sum, the 2-PtIr electrode suffered from the small ECSA compared to the discussed catalysts above, although the Pt:Ir ratio was 99.77:0.23, meaning that the Ir content influenced the ORR performance of the electrode less. Nevertheless, the 2-PtIr electrode performance may suffer from high Pt loading, leading to less accessible sites due to the self-covering of Pt agglomerate structures. In contrast, the above discussed nano scaled catalysts are dispersed onto the support structures, offering a higher number of accessible sites and therefore higher ECSA values than the 2-PtIr electrode.

#### 3.2.2. OER-Activity Results for Two-Step Deposited PtIr Electrodes

Based on the cyclic voltammogram of the 2-PtIr electrode, the voltametric charge was calculated to characterize the OER activity. The voltametric charge (Q_V_) was calculated by numerical integration in the potential range of 0.4–1.4 V vs. RHE, following the work of Rakousky [63]. The calculated Q_V_ of 52.7 mC cm_geo._^−2^ for the 2-PtIr electrode showed more promising OER activity compared to the Q_V_ of 3.8 mC cm_geo._^−2^ of the reference Ir-disc. No further conversion for the obtained Q_V_ was performed in the presented study due to the undefined contributions of the pseudo-capacity of the Ir surface, according to Rakousky [63]. Compared to the Q_V_ values for Ir-based catalysts reported in the literature (approximately 75 mC cm_geo._^−2^ for Ir_0.3_Ti_0.7_O_2_ [64] and approximately 90 mC cm_geo._^−2^ for IrO_2_ [65]), the 2-PtIr electrode showed lower activity towards OER, although the catalyst loading was approximately two-times higher. This result may be linked to the high Pt ratio in the two-phase composition of the 2-PtIr electrode since Pt is recognized to be a poor OER catalyst. 

**Figure 9:**
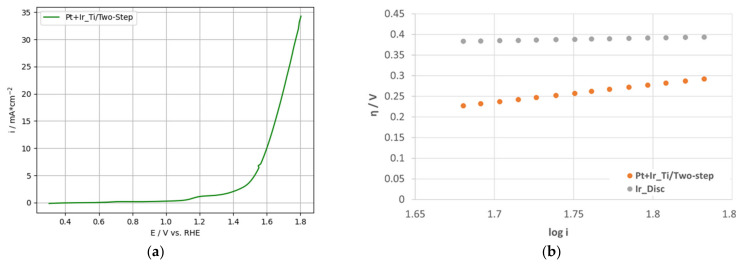
(**a**) Linear voltammetry for two-step deposited PtIr electrode and (**b**) OER Tafel plots for two-step deposited PtIr electrode and polished, high purity Ir-disc.

To further characterize the OER activity of the electrode, LSV measurements (Figure 9a) were performed and analyzed regarding the mass activity (MA) and kinetic parameters. LSV measurements for the Ti-based single Pt electrodes were not performed due to the poor OER performance of Pt-catalysts discussed in the literature [10,22,66].

To determine the MA, the current density at 1.6 V vs. RHE obtained from the LSV measurements was divided by the total catalyst loading (see Table 3) of the electrode [37,63]. The voltage of 1.6 V was chosen because of the cell voltage applied during in-situ tests, ranging from 1.4 V to 1.85 V [63]. 

In addition, the calculation of the kinetic OER parameters was performed based on the LSV measurements according to Tahir et al. [67] and Yan et al. [68]. The kinetic values are summarized in Table 4; the corresponding Tafel plots are shown in Figure 9b supplemented by the Ir-disc Tafel parameters as reference. 

Considering the total catalyst loading, the MA for the 2-PtIr electrode was poor, resulting in 5 A g_Pt + Ir_^−1^. This can be explained by the high Pt loading of the electrode, which made only a small contribution to the OER activity [39]. Similarly, for the MAs reported by Zhang et al. [37], higher Pt incorporation into the catalyst layer corresponded to lower MA activity. Therefore, the 2-PtIr electrode was outperformed by the MAs results based on the total catalyst loading reported by Zhang et al. [37] ranging approximately from 180 A g_Pt + Ir_^−1^ to 290 A g_Pt + Ir_^−1^, and the results of Du et al. [39], ranging approximately from 125 A g_Pt + Ir_^−1^ to 200 A g_Pt + Ir_^−1^. 

The Tafel slope (b) of 428 mV dek^−1^ for the prepared 2-PtIr electrode was nearly six-times higher than the slope for the reference Ir-disc, implying a lower charge transfer factor on the catalyst surface. Further, Kong et al. reported Tafel slopes of 81.3 mV dek^−1^ and 71.1 mV dek^−1^ for chemically reduced Pt on commercial and self-synthesized IrO_2_ catalyst particles which were nearly six-times lower than the value reported here [62]. The lower the Tafel-slope, the faster the charge transfer across the catalyst surface [69]. Additionally, a higher Tafel slope leads to a higher overpotential if the current density is increased during operation. The increased Tafel slope of the prepared 2-PtIr electrode in comparison to the high purity Ir reference disc, as well as the literature data (obtained with glassy carbon RDE), may be linked to polarisation losses of the Ti-substrate. The fiber-based structure can lead to a contact resistance increase and thus increased polarisation losses. 

The exchange current density (i_0_) of 14.1 A m^−2^ for the 2-PtIr electrode was five orders of magnitude higher than that for the Ir-disc reference (Table 4). I_0_ describes the intrinsic activity of the catalyst under equilibrium states. High exchange current densities are needed for OER catalysts, resulting in high electrochemical conversion rates [68]. The significant difference between the exchange current densities discussed here can be linked to the different surface structures. The Ir-disc surface was polished for an even and clean surface, and, therefore, the electrochemical surface area was, in good approximation, equal to the geometrical surface area. In contrast, the catalyst structure of the 2-PtIr electrode was rough and micro- and nanostructured, enabling a high number of electrochemical active sites, although the projected area was equal to the Ir-disc reference. This is also implied by the calculated Q_Desorp_ values based on the cyclic voltammograms. Moreover, the high catalyst loading, combined with the micro and nanostructured catalyst layer, enabled the formation of more electrochemical active sites, leading to enhanced i_0_ values.

### 3.3. Ti-Based Electrodes with One-Step Deposited PtIr Catalyst

Additional experiments were performed to prepare a bifunctional oxygen electrode by the electrochemical deposition of PtIr catalyst in a one-step deposition method using a binary alloy electrolyte. 

**Figure 10:**
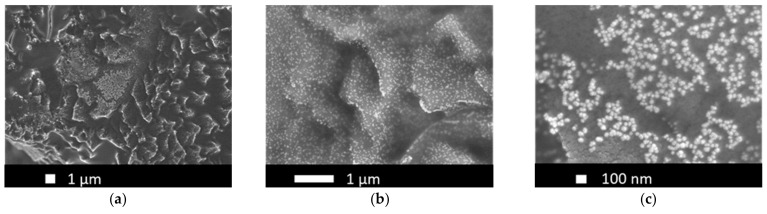
SEM-micrographs of one-step electrodeposited PtIr electrodes at (**a**) 5000×, (**b**) 20,000× and (**c**) 50,000× magnification.

The SEM-micrographs in Figure 10 reveal topographical modifications caused by the wet-chemical pre-treatment step, while Figure 10b, in particular, shows the homogenous distribution of PtIr agglomerates across the modified Ti-fiber surface area. Moreover, an agglomeration of PtIr catalyst can be identified on the Ti-edges resulting from wet-chemical pre-treatment. This finding can be explained by the concentration of electric field lines during electrochemical catalyst deposition on the Ti-edges. The high-resolution SEM-micrograph (Figure 10c) indicates under 100 nm size PtIr agglomerates in a spherical shape on the Ti-fiber surface. The well-dispersed nano-agglomerates are beneficial for the creation of a high electrochemical surface area, thus enhancing electrode performance.

The difference in the catalyst structure obtained for one-step PtIr electrodes (1-PtIr electrodes) can be attributed to the applied method for electrochemical catalyst deposition. For the one-step deposition, only PED was applied, leading to the growth of more nuclei, while agglomerates grew less in size. In contrast, the areal, microsized sub-structure linked to the CPD method for single Pt and 2-PtIr electrodes was not observed for the one-step deposited PtIr electrodes (as shown in Figure 10).

**Figure 11:**
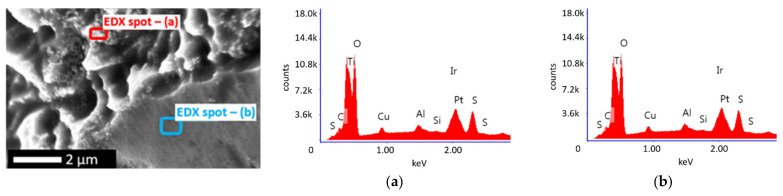
EDX spot analysis of one-step deposited PtIr electrode showing: (**a**) EDX spectrum obtained for the modified surface spot by pre-treatment and (**b**) EDX spectrum for a less modified surface spot.

Comparing the EDX-spectra in Figure 11a,b, it can be seen that in the area of modified Ti a higher count of Pt and Ir is presented than in the spectrum obtained from the less modified area, supporting the identified catalyst distribution based on the SEM micrographs. Due to the proximity of the characteristic X-ray energy of Pt and Ir (2.048 keV for the M-shell of Pt and 1.977 keV for the M-shell of Ir), a differentiation of the counts by elements obtained in the 2 keV region of the spectrum needs to be handled with caution.

The sulfur peak in Figure 11b originated from the previous electrolyte used for the electrochemical half-cell characterization of the electrode. Although the electrodes were cleaned with deionized water after characterization, sulfur residues may have remained on the electrode surface.

Further, the obtained EDX spectrum indicates that Pt and Ir were present on the Ti-substrate, meaning that both elements were successfully deposited. Nevertheless, the EDX analysis cannot provide information regarding the alloy/non-alloy-stage of the PtIr catalyst, only its elemental composition. The one-step deposition was performed using a binary-alloy electrolyte according to Sheela et al. [70]. The authors demonstrated PtIr catalyst deposition in an alloyed composition by applying PED method [70]. Therefore, it was assumed that a PtIr-alloy may also be present in the electrodes examined in this study.

The specific catalyst loading and ratio was determined via ICP-OES. The results are listed in Table 5 implying a weight-related Pt:Ir catalyst ratio of approximately 44:56 wt.%. The catalyst ratio corresponds, in good approximation, to the alloy composition aimed for by the electrolyte manufacturer.

#### 3.3.1. ORR-Activity for One-Step Deposited PtIr Electrodes

**Figure 12:**
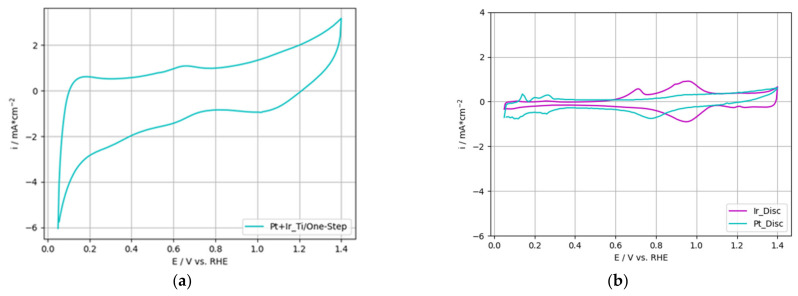
Cyclic voltammogram of (**a**) Ti-based one-step deposited PtIr electrode and (**b**) polished, high purity Pt- and Ir-disc (also shown in inset).

Figure 12a presents the cyclic voltammogram recorded for the 1-PtIr electrode; Figure 12b depicts the voltammograms of polished, high purity Ir- and Pt-disc as reference. For a more detailed view of the Pt- and Ir-disc, refer to the insight voltammogram in Figure 5 and Figure 8. Compared to the references, the specific current densities measured for the 1-PtIr electrode in the entire potential range were higher. 

In the region of hydrogen desorption (0.05 to 0.3 V vs. RHE), no distinct peaks for specific oxide transition states were recorded for the 1-PtIr electrode. Therefore, the cyclic voltammogram indicated a flat hydrogen peak in the entire hydrogen desorption region. This can be linked to the low catalyst loading and balanced PtIr ratio of 44:56 wt.%. Similar results were reported by Jung et al. [40] for a PtIr catalyst consisting of a Pt:Ir ratio of 40:60 wt.%. In addition, it was reported that adding more than 15 wt.% of Ir to the PtIr catalyst layer led to a decrease in the hydrogen desorption peaks [40].

To characterize the ORR-activity, the ECSA was calculated according to the previously discussed procedure for 2-PtIr electrodes. The results are listed in Table 5. The numerical integration of the hydrogen desorption peaks resulted in a poor Q_Desorp_ value and, therefore, to a low SRF of 0.4 cm_echem._^2^ cm_geo._^−2^. Normalized to the catalyst loadings, ECSA resulted in 1.3 m_echem._^2^ g^−1^. This can be attributed to the flattened hydrogen desorption peaks due to the catalyst loading and ratio. 

Du et al. reported ECSAs of 85.3 m_echem._^2^ g_Pt_^−1^ and 75.1 m_echem._^2^ g_Pt_^−1^ for PtIr alloy nanoparticles immobilized onto a carbon support with a Pt:Ir ratio of 20:20 wt.% and 20:40 wt.%, respectively [39]. These results outperformed the 1-PtIr electrode regarding the ECSA obtained in this investigation. In addition, the state-of-the-art Pt/C ORR catalyst, exhibiting an ECSA of 60 m_echem._^2^ g^−1^ to 80 m_echem._^2^ g^−1^, outperformed the characterized 1-PtIr electrode in terms of ORR activity [39,54]. In contrast, Ioroi et al. performed a chemical synthesis of alloyed PtIr particles, determining ECSA values of 7.4 m_echem._^2^ g^−1^, 6.1 m_echem._^2^ g^−1^ and 4.4 m_echem._^2^ g^−1^ for Pt:Ir ratios of 95:5 wt.%, 78:22 wt.% and 63:37 wt.%, respectively [53]. This result indicates that higher incorporation of Ir leads towards a decrease in ORR activity since Ir is a less suitable electrocatalyst for ORR. Further, Garcia et al. synthesized alloyed PtIr particles supported on TiC, TiCN and C via an ethylene glycol method [71]. SRF values of 5.3 cm_echem._^2^ cm_geo._^−2^ and 1.9 cm_echem._^2^ cm_geo._^−2^ were reported for the PtIr/C and PtIr/TiCN catalyst, respectively [71]. Since the PtIr particles were of the same size, the difference in the SRF values was linked to the less homogenous catalyst distribution on the Ti-based support structure [71]. 

To summarize, the 1-PtIr electrode was outperformed by alloyed carbon-based PtIr catalysts. These are not suitable for application as oxygen electrodes due to the carbon corrosion, although they are more electrically conductive than Ti-based substrates. Further, the carbon support enables a more homogenous distribution of the catalysts compared to a Ti-based substrate, leading to more accessible active sites and therefore increased ORR activity. To improve the ORR activity of the 1-PtIr electrode the substrate itself, or the pre-treatment, should be revised, and higher catalyst loadings may be required, since, up to now, only the modified areas of the Ti-fibers are covered with PtIr agglomerates (see Figure 10). 

#### 3.3.2. OER-Activity Results for One-Step Deposited PtIr Electrodes

Based on the cyclic voltammogram from Figure 12a, the Q_V_ value was calculated according to the procedure described in 3.2.2 for 2-PtIr electrodes. The 1-PtIr electrode reached 14.1 mC cm_geo._^−2^, a three-times higher value than for the Ir reference, indicating a higher electrochemical OER activity. Compared to the Q_V_ of 7.3 mC cm_geo._^−2^ for commercial IrO_2_-TiO_2_ particles, characterized via glassy carbon RDE with an increased electrode loading of 100 µg cm^−2^, the 1-PtIr electrode showed a two-times higher value [63]. In contrast, Q_V_ values of approximately 75 mC cm_geo._^−2^ and 90 mC cm_geo._^−2^ for Ir_0.3_Ti_0.7_O_2_ and IrO_2_ on Ti-foil were reported in the literature [64,65]. On the basis of these values, the 1-PtIr electrode was outperformed. Taking the three to five orders of magnitude higher catalyst loadings of 1.3 mg cm^−2^ to 1.7 mg cm^−2^ [64] and 1 mg cm^−2^ [65] into account, the 1-PtIr electrode outperformed the discussed catalysts. Although a high Pt ratio was incorporated into the catalyst layer, being defined as a poor OER catalyst, the 1-PtIr electrode showed increased Q_V_ compared to the single phase Ir catalysts. 

**Figure 13:**
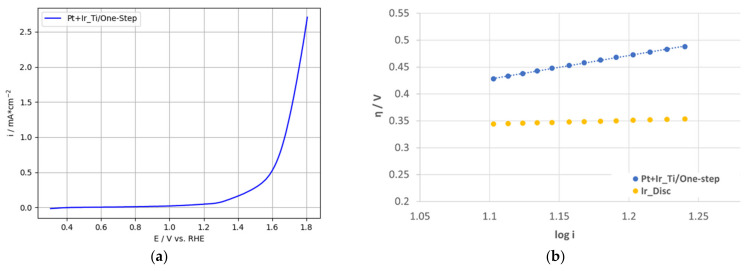
(**a**) Linear voltammetry for one-step deposited PtIr electrode and (**b**) OER Tafel plots for two-step deposited PtIr electrode and polished high-purity Ir-disc.

The kinetic parameters for OER were determined based on the recorded LSV measurements shown in Figure 13a. MA normalized to the specific catalyst loading was calculated according to the previously described procedure (see Table 6). Further, the Ir specific MA was calculated by normalization of the current density to the specific Ir loading (see Table 6), considering Ir as the active catalyst for the OER in this two-phase configuration, following the work of Zhang et al. [37], Du et al. [39] and Silva et al. [72]. Tafel parameters were calculated according to the procedures described previously for 2-PtIr electrodes and listed in Table 6. Tafel plots are depicted in Figure 13b.

The Ir-based MA of the 1-PtIr electrode was more than one order of magnitude lower than values reported for Ir or IrO_2_, though the IrO_2_-TiO_2_ catalyst showed a six times higher MA [63]. The 1-PtIr electrode with a PtIr ratio of 44:56 wt.% was outperformed by the MA of Ir@Pt nanodendrites, with an almost identical PtIr composition of 43:57 wt.%, synthesized and characterized by Zhang et al. [37]. The authors reported an MA of approximately 330 A g_Ir_^−1^ or 187.4 A g_Pt + Ir_^−1^, approximately one order of magnitude higher values compared to the 1-PtIr electrode. This significant difference may be linked to the different nanostructures of the PtIr catalysts. The nanosized Ir@Pt dendritic structure possibly offered more active sites for OER than the nanosized compact spherical PtIr agglomerates obtained in this study [37]. Further, the phase composition of the catalyst systems were different. The PtIr in 1-PtIr electrodes was mixed in the single nanosized catalyst agglomerates; in contrast, the nanodendrites induced a PtIr phase separation, offering higher accessibility to the electrochemical active sites. Moreover, it should be noted that the MAs reported by Zhang et al. [37] were recorded using a very conductive, glassy carbon support as the working electrode in a three-electrode configuration, solely characterizing the catalyst. In this study, we performed the investigations applying a ready-to-use Ti-based electrode, as such being closer to the in-situ application. Nevertheless, the ready-to-use 1-PtIr electrode was competitive with the Pt/IrO_2_ catalyst synthesized via formic acid synthesis by Silva et al. [72]. They reported an Ir-specific MA of 25 A g_Ir_^−1^, 20.3 A g_Ir_^−1^ and 18.8 A g_Ir_^−1^ for atomic Pt:Ir ratios of 1:9, 3:7 and 1:1, respectively, characterized via glassy carbon RDE at a slightly lower voltage of 1.55 V vs. RHE [72]. Furthermore, Kong et al. reported an MA of 29 A g_Ir_^−1^ for a Pt:Ir catalyst (molar ratio of 1:1) prepared via the chemical reduction method, calculated based on a voltage of 1.55 V vs. RHE [62]. Therefore, the MA of 34.4 A g_Ir_^−1^, determined for the 1-PtIr electrode in the present study, is competitive and outperforms the catalysts of Silva et al. [72] and Kong et al. [62], although a Ti-fiber-based substrate was applied. 

The kinetic parameters determined from the LSV measurements listed in Table 6, presented a higher Tafel slope and higher exchange current density for the1-PtIr electrode compared to the Ir reference. Moreover, although the MA for Pt:Ir catalyst particles (molar PtIr ratio 1:1) reported by Kong et al. was lower than for the 1-PtIr electrode, as discussed above, the Tafel slope of these particles was nearly six-times higher than for the 1-PtIr electrode [62]. The higher Tafel slope indicates a lower charge transfer across the catalyst and, therefore, the electrode. This may be linked to the low catalyst loading of the 1-PtIr electrode and the less electrically conductive fiber-based Ti-substrate. The latter led to higher electrical resistance compared to the metallic high-purity Ir disc or glassy carbon RDE applied by Kong et al. [62]. The i_0_ of the 1-PtIr electrode was nearly four orders of magnitude higher than for the Ir reference which may be linked to the different catalyst structures. The polished Ir reference offered nearly the geometrical surface area (1 cm^2^) for the electrochemical reactions, so that only the surface atoms participated in the reaction. In contrast, the 1-PtIr electrode offered a higher quantity of catalyst sites for the reaction, due to the nanosized and homogeneously distributed PtIr spherical agglomerates. 

## 4. Conclusions

An electrochemically active Ti-based bifunctional electrode, with a possible direct in-situ deployment, was successfully developed via one- and two-step electrochemical deposition methods using Pt and Ir as catalysts. Two oxide-stripping methods for the electrode surface were investigated. The wet-chemical pre-treatment of the fiber-based Ti-substrate with oxalic acid resulted in a rougher surface topography compared to physical Ar plasma etching and was chosen as the pre-treatment method for the Ti-substrate.

The highly porous, electrically conductive, and corrosion resistant Ti-substrate may be suitable to overcome current challenges of URFC electrodes, as it allowed the formation of electrochemically active nano- and microsized Pt and PtIr agglomerates. Spectroscopical mappings showed homogenously distributed noble metal catalysts performing competitively for both electrochemical reactions of interest. 

Further, it was observed that the Pt:Ir ratio influenced the electrochemical characterization results. Higher Pt content led to increased hydrogen desorption peaks, while higher Ir incorporation flattened and broaded the hydrogen desorption peaks in cyclic voltammograms. The normalization of the electrochemical results to the catalyst loading of the electrodes is highly important to enable comparisons to the existing literature. Higher Tafel slopes and exchange current densities for OER were determined in both approaches in comparison to the highly pure Ir-disc, which was related to the fiber structure of the Ti-substrate. The OER mass activity of the one-step deposited PtIr electrode was competitive with values reported in literature, due to the low catalyst loading and homogeneously distributed nanosized PtIr agglomerates obtained via the electrochemical deposition method. In contrast, the two-step deposited electrode showed lower mass activity for OER due to the high Pt incorporation of the electrode.

In essence, the electrode developed with the two-step method where Pt and Ir were deposited consecutively showed the following: a Pt:Ir ratio of 99.77:0.23 wt.%; a Tafel slope of 428 mV dek^−1^ and an exchange current density of 14.1 A m^−2^; and a mass activity of 5 A g_Pt + Ir_^−1^ determined at 1.6 V vs. RHE. The co-deposition of PtIr via the one-step method revealed: a Pt:Ir ratio of 44:56 wt.%; a Tafel slope of 436 mV dek^−1^ and an exchange current density of 1.3 A m^−2^; and a mass activity of 19.2 A g_Pt + Ir_^−1^, 34.4 A g_Ir_^−1^ determined at 1.6 V vs. RHE.

The results of the research suggest an industrially meaningful process route, concentrating on a ready-to-use architecture where the electrode investigations included the interaction between the catalyst and the electrode substrate. 

## Figures and Tables

**Table 1:** Deposition parameters for successive PtIr deposition (two-step) and PtIr co-deposition (one-step).

		CPD		PED			
	Noble Metal	U/V vs. CE	t/s	i/mA cm^−2^	t_on_/s	t_off_/s	Cycles
Two-step deposition	Pt	1.8	450	40	0.01	0.056	4500
	Ir	/	/	30	0.025	0.15	3000
One-step deposition	PtIr	/	/	0.4	0.01	0.056	18,000

**Table 2:** Physical and electrochemical characterization results for the Ti-based electrodes with single Pt catalyst.

	Q_Desorp_/ µC cm_geo._^−2^	SRF/ cm_echem._^2^ cm_geo._^−2^	m_Pt_/ µg cm_geo._^−2^	ECSA/ m_echem._^2^ g^−1^
Pt_Ti/CPD_PED_1	6104.4	29.1	262.5	11.1
Pt_Ti/CPD_PED_2	4265.1	20.3	197.8	10.3
Pt_Ti/CPD_PED_3	4825.8	23	219	10.5

Q_Desorp_—hydrogen desorption charge, SRF—surface roughness factor, m_Pt_—mass of Pt loading, ECSA—electrochemical surface area.

**Table 3:** Physical and electrochemical ORR characterization results for the Ti-based electrode with two-step deposited PtIr catalyst.

	Q_Desorp_/ µC cm_geo._^−2^	SRF/ cm_echem._² cm_geo._^−2^	m_Pt_/ µg cm_geo._^−2^	m_Ir_/ µg cm_geo._^−2^	ECSA/ m_echem._^2^ g^−1^
Pt + Ir_Ti/Two-Step	10657.1	50.7	2470.3	5.7	2.1

Q_Desorp_—hydrogen desorption charge, SRF—surface roughness factor, m_Pt_—mass of Pt loading, m_Ir_—mass of Ir loading, ECSA—electrochemical surface area.

**Table 4:** Mass activity and Tafel parameters for the two-step deposited PtIr electrode and polished, high purity Ir-disc.

			Tafel Parameter	
	i@ 1.6 V vs. RHE/ mA cm_geo._^−2^	MA/ A g_Pt + Ir_^−1^	b/ mV dek^−1^	i_0_/ A m^−^²
Pt + Ir_Ti/Two-Step	9.885	5	428	14.1
Ref. Ir-Disc	/	/	68	1.1 × 10^−4^

**Table 5:** Physical and electrochemical ORR characterization results for the Ti-based electrode with one-step deposited PtIr catalyst.

	Q_Desorp_/ µC cm_geo._^−2^	SRF/ cm_echem._^2^ cm_geo._^−2^	m_Pt_/ µg cm_geo._^−2^	m_Ir_/ µg cm_geo._^−2^	ECSA/ m_echem._^2^ g^−1^
Pt + Ir_Ti/One-Step	74.9	0.4	12	15.2	1.3

Q_Desorp_—hydrogen desorption charge, SRF—surface roughness factor, m_Pt_—mass of Pt loading, ECSA—electrochemical surface area.

**Table 6:** Mass activity and Tafel parameters for the one-step deposited PtIr electrode and polished, high purity Ir-disc.

				Tafel Parameter	
	i@ 1.6 V vs. RHE/mA cm_geo._^−2^	MA/ A g_Ir_^−1^	MA/ A g_Pt + Ir_^−1^	b/ mV dek^−1^	i_0_/ A m^−^²
Pt + Ir_Ti/One-Step	0.523	34.4	19.2	436	1.32
Ref. Ir-Disc	/	/	/	68	1.1 × 10^−4^

## Data Availability

The data reported in this study are available from the authors upon request.
